# Tranexamic acid for hyperacute primary IntraCerebral Haemorrhage (TICH-2): an international randomised, placebo-controlled, phase 3 superiority trial

**DOI:** 10.1016/S0140-6736(18)31033-X

**Published:** 2018-05-26

**Authors:** Nikola Sprigg, Katie Flaherty, Jason P Appleton, Rustam Al-Shahi Salman, Daniel Bereczki, Maia Beridze, Hanne Christensen, Alfonso Ciccone, Ronan Collins, Anna Czlonkowska, Robert A Dineen, Lelia Duley, Juan Jose Egea-Guerrero, Timothy J England, Kailash Krishnan, Ann Charlotte Laska, Zhe Kang Law, Serefnur Ozturk, Stuart J Pocock, Ian Roberts, Thompson G Robinson, Christine Roffe, David Seiffge, Polly Scutt, Jegan Thanabalan, David Werring, David Whynes, Philip M Bath

**Affiliations:** a Stroke Trials Unit, Division of Clinical Neuroscience, University of Nottingham, City Hospital Campus, Nottingham, UK; b Stroke, Nottingham University Hospitals NHS Trust, City Hospital Campus, Nottingham, UK; c Centre for Clinical Brain Sciences, University of Edinburgh, Edinburgh, UK; d Department of Neurology, Semmelweis University, Budapest, Hungary; e The First University Clinic of Tbilisi State Medical University, Tbilisi, Georgia; f Department of Neurology, Bispebjerg and Frederiksberg Hospital, University of Copenhagen, Copenhagen, Denmark; g Neurology Unit, Azienda Socio Sanitaria Territoriale di Mantova, Mantua, Italy; h Stroke Service, Adelaide and Meath Hospital, Tallaght, Ireland; i 2nd Department of Neurology, Institute of Psychiatry and Neurology, Warsaw, Poland; j Radiological Sciences, Division of Clinical Neuroscience, University of Nottingham, Queens Medical Centre Campus, Nottingham, UK; k NIHR Nottingham Biomedical Research Centre, Nottingham, UK; l Nottingham Clinical Trials Unit, University of Nottingham, Queen's Medical Centre, Nottingham, UK; m UGC de Medicina Intensiva, Hospital Universitario Virgen del Rocío, Instituto de Biomedicina de Sevilla, Consejo Superior de Investigaciones Científicas, Universidad de Sevilla, Seville, Spain; n Vascular Medicine, Division of Medical Sciences and Graduate Entry Medicine, University of Nottingham, Royal Derby Hospital Centre, Derby, UK; o Department of Clinical Sciences, Danderyd Hospital, Karolinska Institutet, Stockholm, Sweden; p Department of Medicine, National University of Malaysia, Kuala Lumpur, Malaysia; q Division of Neurosurgery, Department of Surgery, National University of Malaysia, Kuala Lumpur, Malaysia; r Department of Neurology, Selcuk University Medical Faculty, Konya, Turkey; s Department of Medical Statistics, London School of Hygiene & Tropical Medicine, London, UK; t Clinical Trials Unit, London School of Hygiene & Tropical Medicine, London, UK; u Department of Cardiovascular Sciences and NIHR Leicester Biomedical Research Centre, University of Leicester, Leicester, UK; v Stroke Research, Faculty of Medicine and Health Sciences, Keele University, Staffordshire, UK; w Stroke Center, Neurology and Department of Clinical Research, University Hospital, University Basel, Basel, Switzerland; x Stroke Research Centre, UCL Institute of Neurology and National Hospital for Neurology and Neurosurgery, University College London, London, UK; y School of Economics, University of Nottingham, University Park, Nottingham, UK

## Abstract

**Background:**

Tranexamic acid can prevent death due to bleeding after trauma and post-partum haemorrhage. We aimed to assess whether tranexamic acid reduces haematoma expansion and improves outcome in adults with stroke due to intracerebral haemorrhage.

**Methods:**

We did an international, randomised placebo-controlled trial in adults with intracerebral haemorrhage from acute stroke units at 124 hospital sites in 12 countries. Participants were randomly assigned (1:1) to receive 1 g intravenous tranexamic acid bolus followed by an 8 h infusion of 1 g tranexamic acid or a matching placebo, within 8 h of symptom onset. Randomisation was done centrally in real time via a secure website, with stratification by country and minimisation on key prognostic factors. Treatment allocation was concealed from patients, outcome assessors, and all other health-care workers involved in the trial. The primary outcome was functional status at day 90, measured by shift in the modified Rankin Scale, using ordinal logistic regression with adjustment for stratification and minimisation criteria. All analyses were done on an intention-to-treat basis. This trial is registered with the ISRCTN registry, number ISRCTN93732214.

**Findings:**

We recruited 2325 participants between March 1, 2013, and Sept 30, 2017. 1161 patients received tranexamic acid and 1164 received placebo; the treatment groups were well balanced at baseline. The primary outcome was assessed for 2307 (99%) participants. The primary outcome, functional status at day 90, did not differ significantly between the groups (adjusted odds ratio [aOR] 0·88, 95% CI 0·76–1·03, p=0·11). Although there were fewer deaths by day 7 in the tranexamic acid group (101 [9%] deaths in the tranexamic acid group *vs* 123 [11%] deaths in the placebo group; aOR 0·73, 0·53–0·99, p=0·0406), there was no difference in case fatality at 90 days (250 [22%] *vs* 249 [21%]; adjusted hazard ratio 0·92, 95% CI 0·77–1·10, p=0·37). Fewer patients had serious adverse events after tranexamic acid than after placebo by days 2 (379 [33%] patients *vs* 417 [36%] patients), 7 (456 [39%] *vs* 497 [43%]), and 90 (521 [45%] *vs* 556 [48%]).

**Interpretation:**

Functional status 90 days after intracerebral haemorrhage did not differ significantly between patients who received tranexamic acid and those who received placebo, despite a reduction in early deaths and serious adverse events. Larger randomised trials are needed to confirm or refute a clinically significant treatment effect.

**Funding:**

National Institute of Health Research Health Technology Assessment Programme and Swiss Heart Foundation.

## Introduction

Spontaneous (non-traumatic) intracerebral haemorrhage is the cause of up to 20% of all strokes, yet accounts for nearly half of all stroke deaths worldwide. Survival after intracerebral haemorrhage has not changed for several decades,^1^ and the only intervention that improves functional outcome is early intensive blood pressure lowering.^2^

Around a quarter of intracerebral haemorrhages are complicated by haematoma expansion, which most often occurs within the first few hours, but can occur at up to 24 h, and is associated with poor outcomes.^3^, ^4^, ^5^ Radiological markers, including the CT angiography (CTA) spot sign, have been used to try to predict which patients are at greater risk of haematoma expansion.^6^ Drug therapies aimed at limiting haematoma expansion include recombinant factor VII, but a meta-analysis of this and other haemostatic therapies found no benefit on functional outcome.^7^

In patients with traumatic haemorrhage (including from head injuries), tranexamic acid, an antifibrinolytic drug, significantly reduces death due to bleeding and all-cause mortality, with no increase in vascular occlusive events.^8^ A post-hoc analysis of the CRASH-2 trial showed that because death due to bleeding occurred early after trauma, timely administration of tranexamic acid was necessary for patients to receive any benefit.^9^ A meta-analysis of tranexamic acid in traumatic intracranial haemorrhage showed that it was associated with a significant reduction in subsequent intracranial bleeding,^10^ and a larger trial is ongoing.^11^ Tranexamic acid also reduced the number of deaths due to bleeding in women with post-partum haemorrhage.^12^ Use of tranexamic acid after acute intracerebral haemorrhage has been tested in two small randomised studies,^13^ including TICH-1,^14^ which assessed the feasibility of a larger trial. The administration of tranexamic acid was feasible and well tolerated.

**Research in context**

**Evidence before this study**

We searched the Cochrane Stroke Trials register, the Cochrane Central Register of Controlled Trials, MEDLINE Ovid, and Embase Ovid for randomised controlled trials of antifibrinolytics and tranexamic acid up to Nov 27, 2017, using the terms “tranexamic acid” and “exp basal ganglia hemorrhage”or “intracranial hemorrhages” or “cerebral hemorrhage” or “intracranial hemorrhage, hypertensive”. To identify further published, ongoing, and unpublished randomised controlled trials we scanned bibliographies of relevant articles and searched international registers of clinical trials in Nov 27, 2017. We searched for trials in all languages. The quality of evidence was assessed with the GRADE approach. We found two small randomised controlled trials of tranexamic acid with a total of 54 participants, with no clear evidence of benefit or harm associated with tranexamic acid. Five further randomised controlled trials are ongoing.

**Added value of this study**

TICH-2 is, to our knowledge, the first large multicentre, international, randomised controlled trial of tranexamic acid in acute spontaneous intracerebral haemorrhage. It included an older population than in previous tranexamic acid trials after trauma and post-partum haemorrhage. Tranexamic acid was not associated with any significant improvement in functional outcome at 90 days, despite a significant reduction in the number of participants with haematoma expansion and fewer deaths by days 2 and 7 among those allocated tranexamic acid. Tranexamic acid was safe, with fewer serious adverse events and no increase in thromboembolic events compared with placebo.

**Implications of all the available evidence**

Although there is insufficient evidence to support the routine use of tranexamic acid in clinical practice for spontaneous intracerebral haemorrhage, the results do not exclude a possible small effect. The reductions in haematoma expansion and early deaths are promising, but larger randomised trials are needed to confirm or refute a clinically significant treatment effect. Future research should also investigate which subgroups of patients are most likely to benefit.

Therefore, the Tranexamic acid for hyperacute primary IntraCerebral Haemorrhage (TICH-2) trial tested the hypothesis that intravenous tranexamic acid reduces death and dependence when given within 8 h of spontaneous intracerebral haemorrhage.

## Methods

### Study design and participants

TICH-2 was an international double-blind, randomised, placebo-controlled, parallel group, phase 3 trial. Participants were enrolled by investigators from acute stroke units at 124 hospital sites in 12 countries: Denmark, Georgia, Hungary, Ireland, Italy, Malaysia, Poland, Spain, Sweden, Switzerland, Turkey, and the UK. Ethics approval was obtained at each site and country before the start of the study. The trial was adopted in the UK by the National Institute Health Research (NIHR) Clinical Research Network and registered as ISRCTN93732214. The full TICH-2 trial protocol and statistical plan have been published.^15^

Adults with acute intracerebral haemorrhage were eligible for inclusion if they were admitted to a participating hospital within 8 h of stroke symptom onset (or time last seen well). We chose 8 h as the treatment window for consistency with both of the previous trials of tranexamic acid in traumatic intracerebral haemorrhage.^9^ We sought to include participants as quickly as possible because haematoma expansion usually occurs in the first few hours after intracerebral haemorrhage,^3^, ^5^ but allowed randomisation up to 8 h because some patients present later and haematoma expansion can occur up to 24 h.^16^, ^17^ Key exclusion criteria were intracerebral haemorrhage secondary to anticoagulation, thrombolysis, trauma, or a known underlying structural abnormality; patients for whom tranexamic acid was thought to be contraindicated; prestroke dependence (modified Rankin Scale [mRS] score >4); life expectancy less than 3 months; and Glasgow Coma Scale score less than 5. The complete list of exclusion criteria has been published previously.^15^

Investigators obtained written informed consent from each participant if they had the capacity to provide it. If participants could not give consent, a relative or representative gave proxy consent. When consent was deferred or given by a proxy, we informed the participant about the trial as soon as possible and sought their consent.

### Randomisation and masking

Randomisation was done centrally in real time. A secure website was used to randomly assign all participants eligible for inclusion to receive tranexamic acid or matching placebo, with 1:1 allocation. The random allocation sequence was generated by the trial programmer. Randomisation was stratified by country, with minimisation for key prognostic factors: age, sex, time since onset, systolic blood pressure, stroke severity on the National Institutes of Health Stroke Scale (NIHSS), presence of intraventricular haemorrhage, and known history of antiplatelet treatment use immediately before stroke onset.

Sharp Clinical Services (Crickhowell, UK) prepared individual masked treatment packs containing four 5 mL glass ampoules of tranexamic acid 500 mg or sodium chloride 0·9%, which were made identical in appearance by the addition of a heat shrink sleeve. Ampoules and the treatment pack were labelled with a unique pack number. Sharp Clinical Services stored the treatment packs and distributed them to pharmacies within trial sites using a web-based system of control. The pharmacy at each participating site received numbered supplies from Sharp Clinical Services. The packs were stored at room temperature and protected from excessive heat and freezing in a restricted access area (more detail is provided in the protocol). The randomisation system allocated each participant a unique number corresponding to a treatment pack containing either tranexamic acid or placebo. Treatment allocation was concealed from all staff and patients involved in the trial.

### Procedures

The intervention, tranexamic acid, was given intravenously as a 1 g loading dose in 100 mL normal saline 0·9% infused over 10 min, followed by another 1 g in 250 mL normal saline 0·9%, which was infused over 8 h. The comparator was a matching placebo (normal saline 0·9%), administered with an identical regimen.

At randomisation, investigators recorded the participants' age, sex, and medical history, as well as their assessment of intracerebral haemorrhage location, intraventricular haemorrhage, and spot sign. Investigators assessed prestroke dependence with the mRS, and stroke severity using the NIHSS and Glasgow Coma Scale. Participants were reviewed at day 2, day 7, and on the day of death or hospital discharge, whichever came first, to gather information on clinical assessment (NIHSS), the process of care measures (eg, blood pressure lowering treatment, neurosurgical intervention), and discharge date and destination (eg, home or institution).

Adherence was assessed by examining the participant's drug chart and recording the trial treatment administered at day 2 (ie, whether all treatment was given, the time and date of the two doses, and any other comments). Adherence was verified by both central review of the drug chart and pharmacies recording returns of residual or unused trial medications.

Central assessors, who were trained and certified in administration of the mRS and masked to treatment allocation, did the final follow-up at 90 days by telephone from the coordinating centre in each country. If the participant or carer could not be contacted, they received a questionnaire covering the same outcome measures by post.

Brain imaging by CT was done as part of routine care before enrolment; a second research CT scan was done after 24 h of treatment to assess haematoma expansion. When multiple scans were done, the scan closest to 24 h after randomisation was used. Central independent expert assessors, who were masked to treatment assignment, assessed CT scans for the location of the intracerebral haemorrhage using a web-based adjudication system. Semi-automated segmentation of the intracerebral haemorrhage was done on Digital Imaging and Communications in Medicine-compliant images to give intracerebral haemorrhage volumes. The user-guided three-dimensional active contour tool^18^ in the ITK-SNAP software (version 3.6) was used for segmentation and one of three assessors did manual editing as required. All assessments were masked to treatment assignment. Haematoma expansion was defined as an absolute increase of more than 6 mL or a relative growth of greater than 33%.

As part of standard routine care, participants received blood pressure lowering treatment, neurosurgery, and venous thromboembolism prophylaxis as appropriate, in accordance with clinical guidelines.

### Outcomes

The primary outcome was functional status at day 90, as assessed with the mRS, which was administered by telephone or postal questionnaire and masked to treatment allocation. This scale is a 7-level ordered outcome ranked as 0 (no symptoms), 1 (no disability despite symptoms), 2 (slight disability but able to look after own affairs), 3 (moderate disability but able to walk without assistance), 4 (moderately severe disability; unable to walk or attend to own bodily needs), 5 (severely disabled; bedridden and requiring constant nursing care), or 6 (death). It is the most commonly used primary outcome in the context of acute stroke trials, and is recommended in international guidelines.

Prespecified secondary outcomes included neurological impairment at day 7 or discharge (whichever came first) assessed with the NIHSS, health-related quality of life measured with EuroQoL-5 dimensions (EQ-5D) health utility status and visual analogue scale, activities of daily living according to the Barthel index, cognition assessed via a modified Telephone Interview for Cognitive Status-modified.(TICS-M) and verbal fluency, mood assessed with the Zung depression scale (ZDS), costs (length of hospital stay and discharge destination), and radiological efficacy (change in haematoma volume from baseline to 24 h and haematoma location). Data on the day 365 outcomes specified in the protocol are still being collected and will be reported separately.

We prespecified subgroups in the statistical analysis plan:^19^ age (<70 years *vs* ≥70 years), sex (female *vs* male), time from onset to randomisation (<3 h *vs* ≥3 h), mean systolic blood pressure (<170 mm Hg *vs* ≥170 mm Hg), stroke severity (NIHSS score <15 *vs* ≥15), presence of intraventricular haemorrhage (no *vs* yes), known history of antiplatelet treatment before stroke onset (no *vs* yes), spot sign on CTA (yes *vs* no), intracerebral haemorrhage location (supratentorial deep *vs* supratentorial lobar), and ethnicity (other *vs* white).

Prespecified safety outcomes were death, venous thromboembolism, ischaemic events (stroke, transient ischaemic attack, myocardial infarction, acute coronary syndrome, peripheral artery disease), and seizures. These were reported up to day 90, along with all serious adverse events in the first 7 days. Safety outcomes and serious adverse events were independently adjudicated masked to treatment assignment. Serious adverse events were categorised in accordance with the medical dictionary for regulatory authorities (MeDRA).

### Statistical analyses

The total sample size based on an ordinal primary analysis was estimated at 2000 participants, assuming significance of 5%, power of 90%, an ordinal odds ratio (OR) of 0·79, a distribution of the mRS based on data from participants with primary intracerebral haemorrhage in the ENOS trial^20^ (4% had mRS 0, 17% had 1, 16% had 2, 19% had 3, 24% had 4, 7% had 5, and 13% had 6), increases due to loss of follow-up at 5%, and a 20% reduction for baseline covariate adjustment. The OR of 0·79 was chosen because it lay in the range seen in related trials.^10^, ^21^ The effect of tranexamic acid on an unfavourable functional status (ie, death, vegetative state, or fully dependent requiring attention day and night or dependent but not requiring constant attention) had a relative risk of 0·77 (95% CI 0·59–1·02) when given within 8 h of traumatic intracerebral haemorrhage.^11^

We followed a prespecified statistical plan^19^ and analysed the primary outcome as a shift in the mRS at 90 days, using ordinal logistic regression with adjustment for the stratification and minimisation criteria. We tested the assumption of proportional odds with the likelihood ratio test. We also did sensitivity analyses of the day 90 mRS without adjustment and as a binary outcome (dichotomised at mRS 0–3 *vs* 4–6). Analyses were done in accordance with the intention-to-treat (ITT) principle, with participants kept in the groups to which they were allocated by the minimisation algorithm. We defined the ITT group as all participants who underwent randomisation. Safety outcomes were also analysed in the ITT population.

We analysed prespecified subgroups using ordinal logistic regression, with adjustment for the stratification and minimisation criteria, to assess the heterogeneity of the treatment effect on the primary outcome. We analysed secondary outcomes with multiple linear regression for continuous outcomes, binary logistic regression for binary outcomes, and Cox proportional hazards regression for time-to-event data. The nominal level of significance for all analyses, including interaction testing, was a p value less than 0·05. No adjustment was made for multiplicity of testing. KF and PS did the statistical analyses in accordance with the published statistical analysis plan using SAS software version 9.4.^19^

As detailed in the statistical analysis plan, any missing minimisation criteria were given the highest risk value in order for that participant to be included in the analyses. For one participant for whom we did not know if they were taking previous antiplatelet therapy before their intracerebral haemorrhage, we assigned the highest risk value, which in this case was yes. No other missing values were imputed. In case treatment was associated with asymmetric effects on death and other outcome measures, an extreme value was used in participants who had died before day 90 for outcomes that do not include death as part of their scale (0 for EQ-5D health utility status, −1 for EQ visual analogue scale, −1 for TICS-M, and 102·5 for ZDS).

The trial was overseen by a trial steering committee, and an international advisory committee consisting of each national coordinator. A trial management committee based at the Stroke Trials Unit in Nottingham, UK, was responsible for day-to-day conduct of the trial. An independent data monitoring committee reviewed the unmasked data every 6 months. Study data were collected, monitored, and analysed in Nottingham. The trial was done in accordance with the principles of good clinical practice and the Declaration of Helsinki.

### Role of the funding source

The funder of the study had no role in study design, data collection, data analysis, data interpretation, or writing of the report. The corresponding author had full access to all the data in the study and had overall responsibility for the decision to submit for publication.

## Results

Recruitment started on March 1, 2013, and ended on Sept 30, 2017, after a 12-month extension was sought and approved to enable the trial to reach its target sample size. This slower than planned recruitment was due to delays in opening trial sites outside the UK. Subsequently, recruitment increased, and the Trial Steering Committee agreed that the study should exceed the target of 2000 and continue until the end of the extension. Therefore, a total of 2325 participants were recruited from 124 sites in 12 countries over 55 months. 1161 participants were randomly assigned to receive tranexamic acid and 1164 to receive placebo.

Most participants were recruited in the UK (1910 [82%] of 2325; appendix). The mean age was 68·9 years (SD 13·8) and 1301 (56%) participants were male (table 1). The median time from stroke onset to randomisation was 3·6 h (IQR 2·6–5·0) and 833 (36%) participants were recruited within 3 h. Mean baseline systolic blood pressure was 173 mm Hg (SD 27·5) and diastolic blood pressure was 93 mm Hg (18·4). 1371 (59%) participants had a haematoma that was deep and supratentorial, whereas 738 (32%) had one that was lobar and supratentorial; 745 (32%) participants had intraventricular haemorrhage. Mean haematoma volume was 24·0 mL (SD 27·2) and median haematoma volume was 14·1 mL (IQR 5·9–32·4). Contrast-enhanced imaging in the form of CTA was done in 249 (11%) participants. Of these individuals, 24 (20%) of 121 in the tranexamic acid group and 32 (25%) of 128 in the placebo group were spot positive. Treatment groups were well balanced at baseline (table 1).

**Table 1:** Baseline characteristics

		**Tranexamic acid (n=1161)**	**Placebo (n=1164)**
Age*, years		69·1 (13·7) [29–97]	68·7 (13·9) [20–101]
	>70	584 (50%)	580 (50%)
Sex*, male		642 (55%)	659 (57%)
Ethnic origin			
	White	986 (85%)	992 (85%)
	Other	174 (15%)	172 (15%)
Onset to randomisation*, h		3·6 (2·6–5·1) [1·0–20·8]	3·7 (2·6–5·0) [0·8–8·0]
	≤3	421 (36%)	412 (35%)
	≤4·5	779 (67%)	796 (68%)
History			
	Previous antiplatelet therapy*	316 (27%)	295 (25%)
	Statin use prior to admission	319 (28%)	303 (26%)
	Previous stroke or transient ischaemic attack	173 (15%)	156 (14%)
	Ischaemic heart disease	110 (10%)	92 (8%)
Prestroke mRS		0 (0–1) [0–4]	0 (0–1) [0–4]
Glasgow Coma Scale		13 (2·2) [5·0–150]	14 (2·1) [5·0–15·0]
NIHSS score*		13 (7·5) [0·0–41·0]	13 (7·5) [0·0–42·0]
Systolic blood pressure*, mm Hg		172 (27·5) [98·0–265]	174 (26·8) [99·0–265]
Diastolic blood pressure, mm Hg		93 (18·4) [46·0–179]	94 (17·8) [35·5–162]
Haematoma location			
	Supratentorial lobar	379 (33%)	359 (31%)
	Supratentorial deep	675 (58%)	696 (60%)
	Infratentorial	73 (6%)	76 (7%)
	Combination	34 (3%)	33 (3%)
Intracerebral haematoma volume (mL)		14·1 (5·9–32·4) [0·0–207]	12·5 (5·1–31·9) [0·0–163]
Intraventricular haemorrhage*		382 (33%)	363 (31%)
CT angiography done		121 (11%)	128 (11%)
	Spot positive	24 (20%)	32 (25%)
	Spot negative	97 (80%)	96 (75%)

Data are n (%), mean (SD), or median (IQR). Values in square brackets are ranges. Baseline information was missing for one participant for ethnic origin, one for history of previous antiplatelet therapy, 18 for history of statin use, 24 for history of previous stroke or transient ischaemic attack, 27 for history of ischaemic heart disease, 29 for history of thromboembolism, 48 for CT angiography, and 52 for haematoma volume. mRS=modified Rankin Scale. NIHSS=National Institutes of Health Stroke Scale.

* Minimisation criteria.

Adherence to per protocol treatment for the allocated treatment was high: 2207 (95%) of 2325 participants received all of their randomised treatment, whereas only 15 (1%) received no treatment (appendix). Adherence did not differ between the groups. The median time from randomisation to treatment was 21 min (IQR 13–33).

**Figure 1:**
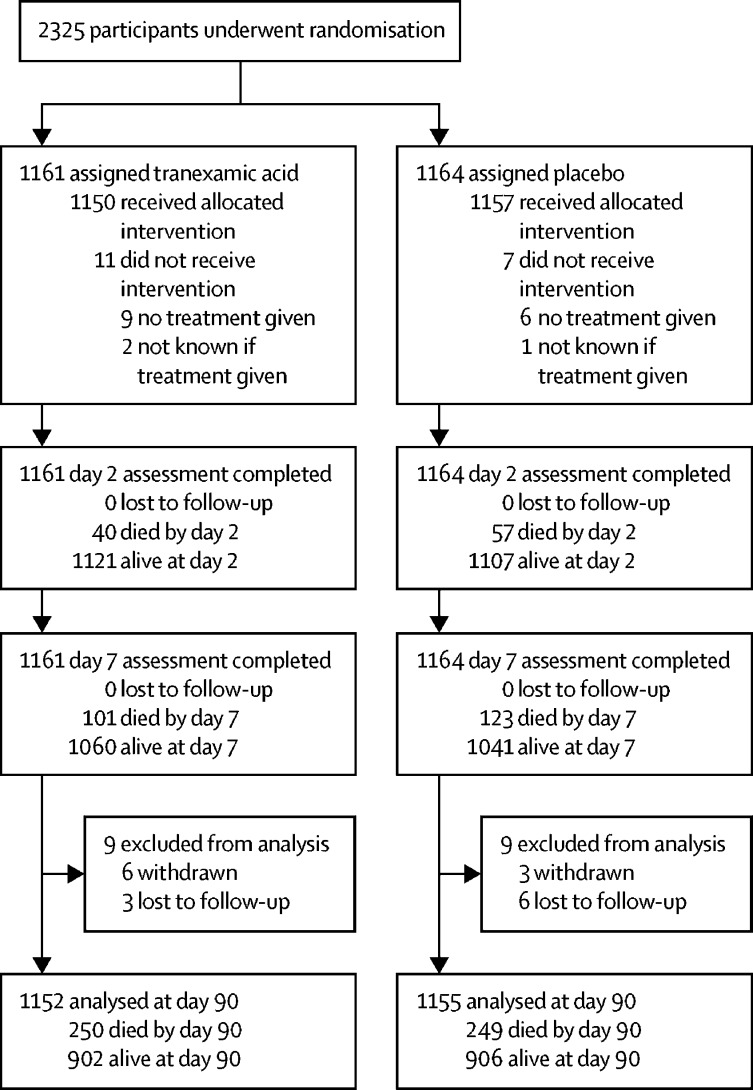
Trial profile

**Figure 2:**
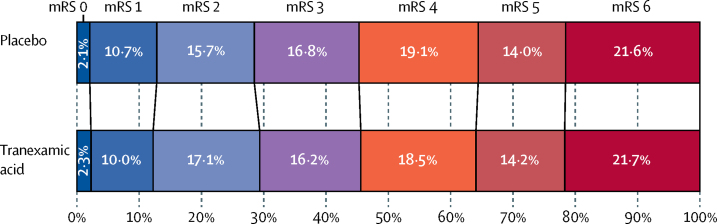
Shift plot of day 90 mRS An mRS score of 0 represents no symptoms, 1 represents no disability despite symptoms, 2 represents slight disability but able to look after own affairs, 3 represents moderate disability but able to walk without assistance, 4 represents moderately severe disability (unable to walk or attend to own bodily needs), 5 represents severely disabled (bedridden and requiring constant nursing care), and 6 represents death. mRS=modified Rankin Scale.

The primary outcome of mRS at day 90 was assessed in 2307 (99%) of 2325 participants; nine (<1%) were lost to follow-up and nine (<1%) withdrew from their day 90 follow-up (figure 1). There was no difference in the distribution (shift) in the mRS at day 90 after adjustment for stratification and minimisation criteria, with an adjusted odds ratio (aOR) of 0·88 (95% CI 0·76–1·03, p=0·11; table 2 and figure 2). A formal goodness-of-fit test showed no evidence that the proportional odds assumption was violated (p=0·97). In a sensitivity analysis, we detected no difference between the groups in the proportion of participants who were dead or dependent at day 90 (mRS >3) and the aOR was 0·82 (95% CI 0·65–1·03, p=0·08; table 2).

**Table 2:** Primary and secondary outcomes

	**Tranexamic acid (n=1161)**	**Placebo (n=1164)**	**Adjusted**	
			Effect estimate (95% CI)	p value
**Primary outcome, day 90**				
Participants with mRS outcome	1152	1155	Ordinal OR 0·88 (0·76 to 1·03)	0·11
mRS 0	26 (2%)	24 (2%)	..	..
mRS 1	115 (10%)	124 (11%)	..	..
mRS 2	197 (17%)	181 (16%)	..	..
mRS 3	187 (16%)	194 (17%)	..	..
mRS 4	213 (18%)	221 (19%)	..	..
mRS 5	164 (14%)	162 (14%)	..	..
mRS 6, death	250 (22%)	249 (22%)	..	..
**Sensitivity analysis, day 90**				
mRS, unadjusted	..	..	Ordinal OR 1·00 (0·86 to 1·15)	0·97
mRS >3	814 (71%)	826 (72%)	Binary OR 0·82 (0·65 to 1·03)	0·08
**Haematoma**				
Change in volume from baseline to 24 h*, mL	3·72 (15·9)	4·90 (16·0)	MD −1·37 (−2·71 to −0·04)	0·0432
Participants with haematoma expansion†	265 (25%)	304 (29%)	Binary OR 0·80 (0·66 to 0·98)	0·0300
**Day 7**				
Death by day 7	101 (9%)	123 (11%)	Binary OR 0·73 (0·53 to 0·99)	0·0406
NIHSS day 7	10·13 (8·3)	10·29 (8·3)	MD −0·43 (−0·94 to 0·09)	0·10
**Day 90**				
Death by day 90	250 (22%)	249 (21%)	HR 0·92 (0·77 to 1·10)	0·37
EQ-5D HUS, out of 1	0·34 (0·4)	0·34 (0·4)	MD 0·01 (−0·01 to 0·04)	0·30
EQ-VAS, out of 100	48·81 (33·8)	48·34 (33·1)	MD 1·75 (−0·52 to 4·02)	0·13
Barthel index, out of 100	52·92 (44·0)	53·21 (43·7)	MD 1·70 (−0·90 to 4·31)	0·20
TICS-M, out of 39	13·57 (12·5)	13·94 (12·8)	MD −0·19 (−1·12 to 0·74)	0·69
ZDS, out of 100	67·28 (29·5)	67·29 (29·9)	MD −0·35 (−2·60 to 1·90)	0·76
Global analysis (Wei-Lachin test)	..	..	MWD 0·00 (−0·05 to 0·04)	0·85
**Discharge information**				
Length of stay in hospital, days	63·12 (47·1)	63·73 (48·1)	MD 1·09 (0·97 to 1·24)	0·16
If discharged, days well at home	69·94 (28·6)	72·15 (29·1)	MD −0·72 (−3·73 to 2·28)	0·64
**Disposition at discharge**				
Home	465 (40%)	453 (39%)	Binary OR 1·14 (0·93 to 1·40)	0·20
Institution	505 (43)	506 (43%)	Binary OR 0·99 (0·83 to 1·18)	0·90
Died by discharge	190 (16%)	205 (18%)	Binary OR 0·83 (0·65 to 1·07)	0·15

Data are n (%) or mean (SD), unless noted otherwise. OR=odds ratio. MD=mean difference. HR=hazard ratio. MWD=Mann-Whitney difference. mRS=modified Rankin Scale. NIHSS=National Institutes of Health Stroke Scale. EQ-5D HUS=EuroQol-5 dimensions health utility status. EQ-VAS=EuroQol visual analogue scale. TICS-m=Telephone Interview for Cognitive Status-modified. ZDS=Zung Depression Scale.

* Adjusted for baseline haematoma volume.

† Haematoma expansion defined as an increase of >6 mL or a growth of >33%.

**Figure 3:**
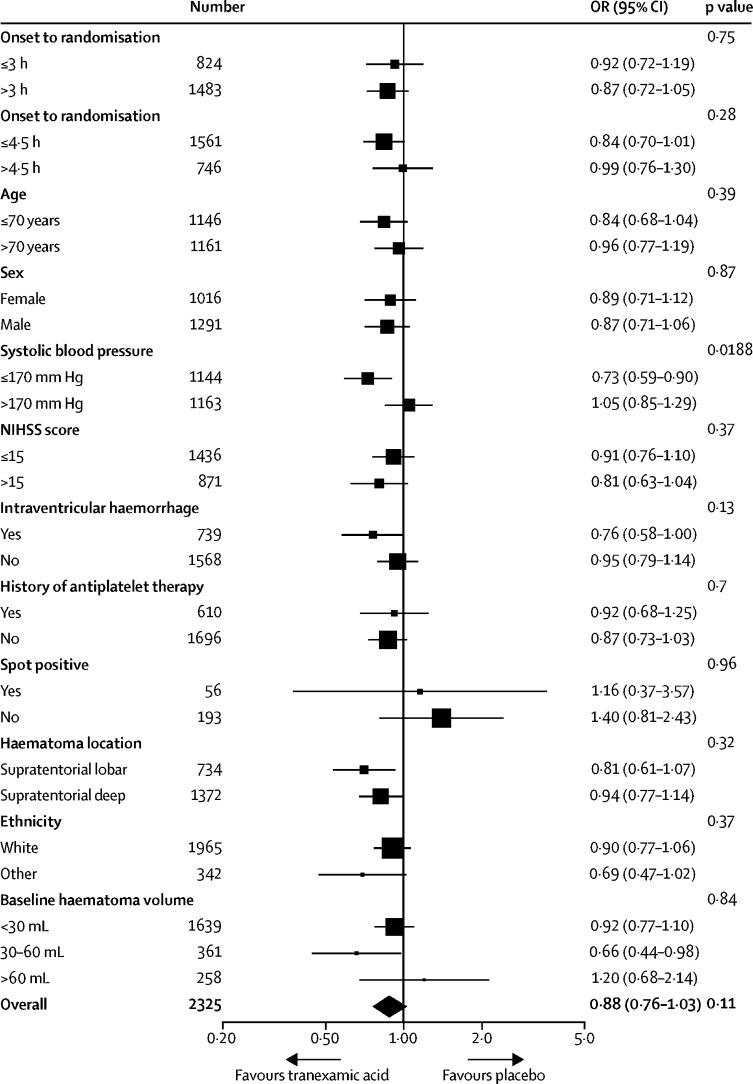
Primary outcome by subgroups All subgroups were predefined except for intracerebral haemorrhage volume, which was added as a post-hoc analysis. OR=odds ratio. NIHSS=National Institutes of Health Stroke Scale.

When the primary outcome was assessed in prespecified subgroups (figure 3), the only significant interaction was between mRS and baseline systolic blood pressure (interaction p=0·0188), such that participants with a baseline systolic blood pressure less than or equal to 170 mm Hg had a favourable shift in mRS with tranexamic acid compared with those with a systolic blood pressure greater than 170 mm Hg. There was no heterogeneity of treatment effect by time of administration (figure 3), whether dichotomised as less than 3 h versus 3 h or longer (interaction p=0·75) or as less than 4·5 h versus 4·5 h or longer (interaction p=0·28); similarly, there was no interaction between treatment effect and time when analysed as a continuous variable (aOR 0·98, 95% CI 0·90–1·07, p=0·69).

Fewer participants had haematoma expansion at day 2 in the tranexamic acid group (265 [25%] of 1054 participants) than in the placebo group (304 [29%] of 1058 participants; aOR 0·80, 95% CI 0·66 to 0·98, p=0·0300). The mean increase in haematoma volume from baseline to 24 h was also less in the tranexamic acid group (3·72 mL, SD 15·9) than in the placebo group (4·90 mL, 16·0; adjusted mean difference −1·37, 95% CI −2·71 to −0·04, p=0·0432). Neurological impairment (mean NIHSS score at day 7) did not differ between the tranexamic acid group and placebo group (adjusted mean difference −0·43, 95% CI −0·94 to 0·09; p=0·10).

There were no significant differences in any of the day 90 functional outcomes between treatment groups—ie, activities of daily living, mood, cognition, or quality of life (table 2). Length of hospital stay and discharge disposition did not differ between treatment groups (table 2).

By day 7, fewer patients had died in the tranexamic acid group (101 [9%] of 1161) than in the placebo group (123 [11%] of 1164; table 2). However, the numbers of deaths by day 90 did not differ between the tranexamic acid group (250 [22%] patients) and the placebo group (249 [21%] patients; table 2). Survival did not differ between the treatment groups over 90 days (adjusted hazard ratio 0·92, 95% CI 0·77–1·10, p=0·37; appendix).

Participants in the tranexamic acid group had fewer predefined safety outcomes and serious adverse events than those in the placebo group at day 2 (379 [33%] patients *vs* 417 [36%] patients, p=0·0272), day 7 (456 [39%] *vs* 497 [43%], p=0·0200), and 90 days (521 [45%] *vs* 556 [48%], p=0·0393; appendix). There was no increase in venous thromboembolic events (39 [3%] patients in the tranexamic acid group *vs* 37 [3%] in the placebo group; p=0·98) or arterial occlusions (myocardial infarction, acute coronary syndrome, or peripheral arterial occlusion) in the tranexamic acid group compared with the placebo group (appendix). Seizure was the most common safety outcome (77 [7%] patients in the tranexamic acid group *vs* 85 [7%] in the placebo group) and nervous system disorders were the most common serious adverse events (149 [13%] *vs* 163 [14%]), followed by infections (98 [8%] *vs* 116 [10%]).

## Discussion

In this trial of tranexamic acid versus placebo after acute intracerebral haemorrhage, there was no significant difference between the groups in the primary outcome of functional status at day 90. However, in the tranexamic acid group, we detected significant reductions in the prespecified secondary outcomes of early death, haematoma expansion, and serious adverse events, consistent with tranexamic acid having an antifibrinolytic effect after intracerebral haemorrhage.

Tranexamic acid was associated with a small but significant reduction in haematoma expansion and smaller haematoma volumes, key factors that are known to affect outcomes after intracerebral haemorrhage.^4^, ^6^ However, the small reduction in haematoma volume (1·37 mL smaller in the tranexamic acid group than in the placebo group) might have been insufficient to translate into improved functional status in this population.

One explanation for our findings could be that the anticipated treatment effect (OR 0·79) was too large, which the trial was unable to detect. Indeed, previous randomised controlled trials of tranexamic acid in other settings have enrolled more than ten times the number of participants to identify smaller effects on bleeding-related deaths after trauma (OR 0·85)^8^ and post-partum haemorrhage (OR 0·81).^12^ Furthermore, the findings of an individual patient data meta-analysis of 40 138 participants have subsequently shown that it is necessary to start tranexamic acid within 3 h of the start of bleeding to receive any benefit in other conditions.^14^

We found no evidence of an increase in serious adverse effects with tranexamic acid; notably, there was no increase in venous thromboembolism in this significantly older population with more comorbidities than participants in previous studies of tranexamic acid.^8^, ^12^ It is therefore unlikely that any potential benefit of tranexamic acid was offset by harm, as has been suggested with recombinant factor VIIa.^22^ In a phase 3 trial, there was no evidence of clinical benefit from recombinant factor VIIa, which was associated with a reduction in haematoma expansion but an increased risk of arterial occlusive events.^22^ Although tranexamic acid and recombinant factor VIIa are both haemostatic agents, tranexamic acid acts through antifibrinolytic mechanisms and recombinant factor VIIa is a procoagulant, so they have different risk-benefit profiles.

To date, the only intervention to improve functional outcome after intracerebral haemorrhage is early intensive blood pressure lowering.^2^ Although no significant effects on haematoma growth were detected in INTERACT-2,^2^ secondary analysis suggested that blood pressure lowering did attenuate bleeding in a dose-dependent manner.^23^ The interaction between baseline systolic blood pressure and treatment in our study suggests that participants with lower blood pressure were more likely to benefit from tranexamic acid. This finding could have been confounded by stroke severity, given that larger haematomas have increased blood pressure and worse outcomes.^21^, ^24^

Baseline haematoma volume is the strongest predictor of outcome after spontaneous intracerebral haemorrhage, and in an exploratory post-hoc analysis, participants with a baseline haematoma volume of between 30 mL and 60 mL who received tranexamic acid seemed to have better outcomes (figure 3). Although this finding could be due to chance, it is also compatible with the notion that patients with moderately sized haematomas might be more likely to benefit from tranexamic acid, and hence could be targeted for future studies, as has been postulated with recombinant factor VIIa.^21^, ^22^

The strengths of this study include its double-blinding, allocation concealment, low risk of bias, high adherence to treatment, and very few missing data on primary outcomes. Treatment groups were well balanced for baseline factors. The use of approved brief and proxy consent processes allowed the rapid enrolment of patients without the capacity to consent, which is important to avoid bias in acute stroke studies. Our inclusion criteria were deliberately broad to reflect the clinical population and to facilitate recruitment from multiple international sites. Nevertheless, most participants were recruited from the UK. The study had several other limitations. We did not collect screening logs so are unable to present data on eligibility. Wide inclusion criteria led to a heterogeneous population with more severe strokes, larger haematoma volumes, and a greater proportion of lobar haematomas and intraventricular haemorrhage than populations in other intracerebral haemorrhage trials,^2^, ^21^, ^22^, ^25^ which could have diluted any potential treatment effect. Finally, despite efforts to ensure rapid treatment, most participants were enrolled more than 3 h after the onset of intracerebral haemorrhage, which could explain the absence of a significant interaction with time in the subgroup analysis.

Several smaller randomised controlled trials of tranexamic acid in intracerebral haemorrhage are ongoing (STOP-MSU [NCT03385928], STOP-AUST [NCT01702636], TRAIGE [NCT02625948], TRANSACT [NCT03044184], and TICH-NOAC [NCT02866838]; total enrolment is fewer than 1000 participants across the trials), and a meta-analysis of individual patient data is planned.^26^ Future trials would need to enrol far more patients to allow the detection of a small treatment effect, or enrich their population with patients at greatest risk of haematoma expansion, primarily by treating patients earlier.^27^ Although patients presenting early are at greater risk of haematoma expansion, they also have more severe strokes and larger haematoma volumes.^24^ Identification of patients most likely to benefit from haemostatic therapy on the basis of factors other than time of onset has been suggested,^28^ but enrichment with the CTA spot sign has yet to be successful.^29^

In summary, tranexamic acid did not affect functional status at day 90, although potential benefits were seen with reductions in haematoma expansion, early death, and serious adverse events. The observed effect size was smaller than anticipated and is compatible with a lack of efficacy or the presence of a smaller treatment effect than expected. Future research should investigate which subgroups of patients might benefit. Tranexamic acid is inexpensive, easy to administer, seems to be safe, and is widely available, so even a modest treatment effect could have an important impact on the global scale. Larger randomised trials are warranted.
