# Supplementary material for: Tranexamic acid for hyperacute primary IntraCerebral Haemorrhage (TICH-2): an international randomised, placebo-controlled, phase 3 superiority trial
Source: Lancet. 2018 May 26;391(10135):2107–15. doi: 10.1016/S0140-6736(18)31033-X (PMC5976950; doi:10.1016/S0140-6736(18)31033-X)
Supplement: Supplementary appendix [file mmc1.pdf]

THE LANCET

Supplementary appendix

This appendix formed part of the original submission and has been peer reviewed. We post it as supplied by the authors.

Supplement to: Sprigg N, Flaherty K, Appleton JP, et al. Tranexamic acid for hyperacute primary IntraCerebral Haemorrhage (TICH-2): an international randomised, placebo-controlled, phase 3 superiority trial. *Lancet* 2018; published online May 16. http://dx.doi.org/10.1016/S0140-6736(18)31033-X.

Supplementary appendix

Supplementary table 1 – Recruitment by country

Country	Participants Recruited N (%)	Average per month
Total number recruited	2325	
United Kingdom	1910 (82·2%)	32·93
Georgia	141 (6·1%)	3·28
Italy	96 (4·1%)	1·92
Malaysia	46 (2·0%)	1·64
Switzerland	46 (2·0%)	1·53
Denmark	39 (1·7%)	2·17
Republic of Ireland	17 (0·7%)	0·65
Hungary	9 (0·4%)	0·64
Turkey	9 (0·4%)	0·33
Sweden	8 (0·3%)	0·29
Poland	3 (0·1%)	0·19
Spain	1 (0·0%)	0·06

Supplementary table 2 – Adherence

	TXA	Placebo	p
Number of participants randomised	1161	1164	
All randomised treatment received as intended, n (%)	1101 (94·8%)	1106 (95·0%)	0·96
Some randomised treatment received, n (%)	49 (4·2%)	51 (4·4%)	0·66
No randomised treatment received, n (%)	9 (0·8%)	6 (0·5%)	0·48
Not known what treatment received, n (%)	2 (0·2%)	1 (0·1%)	0·54
Protocol violation submitted for incorrect drug administration*, n (%)	31 (2.7%)	26 (2.2%)	0.56
Time until first dose (minutes), median [IQR] {range}	21 [13, 33] {1, 167}	21 [13, 32] {1, 196}	0·49

*Protocol violations were investigator reported. Data are number (%), median [IQR].

Supplementary table 3 – Serious adverse event information

	Median days to event [IQR]	TXA 1161	Placebo 1164	p
By day 2		379 (32·6%)	417 (35·8%)	0·0272
By day 7		456 (39·3%)	497 (42·7%)	0·0200
By day 90		521 (44·9%)	556 (47·8%)	0·0393
**Safety outcomes**				
Death	1 [0, 15]	250 (21·5%)	249 (21·4%)	0·60
ACS or MI	5 [1, 53]	11 (0·9%)	6 (0·5%)	0·24
Deep vein thrombosis (DVT)	15 [10, 34]	19 (1·6%)	14 (1·2%)	0·41
Pulmonary embolism (PE)	16 [8, 29]	20 (1·7%)	23 (2·0%)	0·58
VTE (combined DVT/PE)	16 [10, 30]	39 (3·4%)	37 (3·2%)	0·98
Seizure / convulsions	1 [0, 3]	77 (6·6%)	85 (7·3%)	0·44
Ischaemic stroke or TIA	9 [2, 52]	16 (1·4%)	11 (0·9%)	0·27
**SAEs by subcategory**				
Blood and lymphatic system disorders	0 [0, 4]	1 (0·1%)	2 (0·2%)	0·41
Cardiac disorders	1 [1, 2]	14 (1·2%)	10 (0·9%)	0·48
Gastrointestinal disorders	1 [0, 2]	12 (1·0%)	9 (0·8%)	0·59
General disorders and administration site conditions	2 [1, 3]	2 (0·2%)	2 (0·2%)	0·95
Immune system disorders	0 [0, 1]	1 (0·1%)	2 (0·2%)	0·51
Infections and infestations	2 [1, 3]	98 (8·4%)	116 (10·0%)	0·14
Injury, poisoning and procedural complications	1 [1, 3]	7 (0.6%)	10 (0.9%)	0.53
Metabolism and nutrition disorders	4 [1, 7]	0	2 (0·2%)	0·99
Musculoskeletal and connective tissue disorders	1 [1, 1]	0	1 (0·1%)	0·99
Neoplasms benign, malignant and unspecified	4 [1, 5]	3 (0·3%)	3 (0·3%)	0·89
Nervous system disorders	1 [0, 1]	171 (14.7%)	189 (16.2%)	0.24
Psychiatric disorders	4 [3, 5]	1 (0·1%)	4 (0·3%)	0·26
Renal and urinary disorders	2 [1, 4]	14 (1·2%)	17 (1·5%)	0·66
Respiratory, thoracic and mediastinal disorders	1 [1, 2]	4 (0·3%)	3 (0·3%)	0·72
Skin and subcutaneous tissue disorders	5 [5, 5]	2 (0·2%)	0	0·99

	Median days to event [IQR]	TXA 1161	Placebo 1164	p
Vascular disorders	1 [0, 2]	7 (0·6%)	9 (0·8%)	0·64
Miscellaneous	1 [0, 3]	7 (0·6%)	6 (0·5%)	0·81

Data are number (%), median [IQR]. This table gives details on the serious adverse events experienced up until each participant's day 7 follow-up; safety outcomes and death are collected up until day 90. The numbers given in each group are per participant, as opposed to the total number of events. Categorisation is according to Medical dictionary for regulatory authorities (MeDRA). The by site section will be given for number of participants in each row; if someone had an event in both the cardiovascular and nervous system categories then they would appear twice, once in each of these categories. Comparisons will be done using binary logistic regression.

Supplementary figure 1 – Plot of cumulative mortality up to 120 days

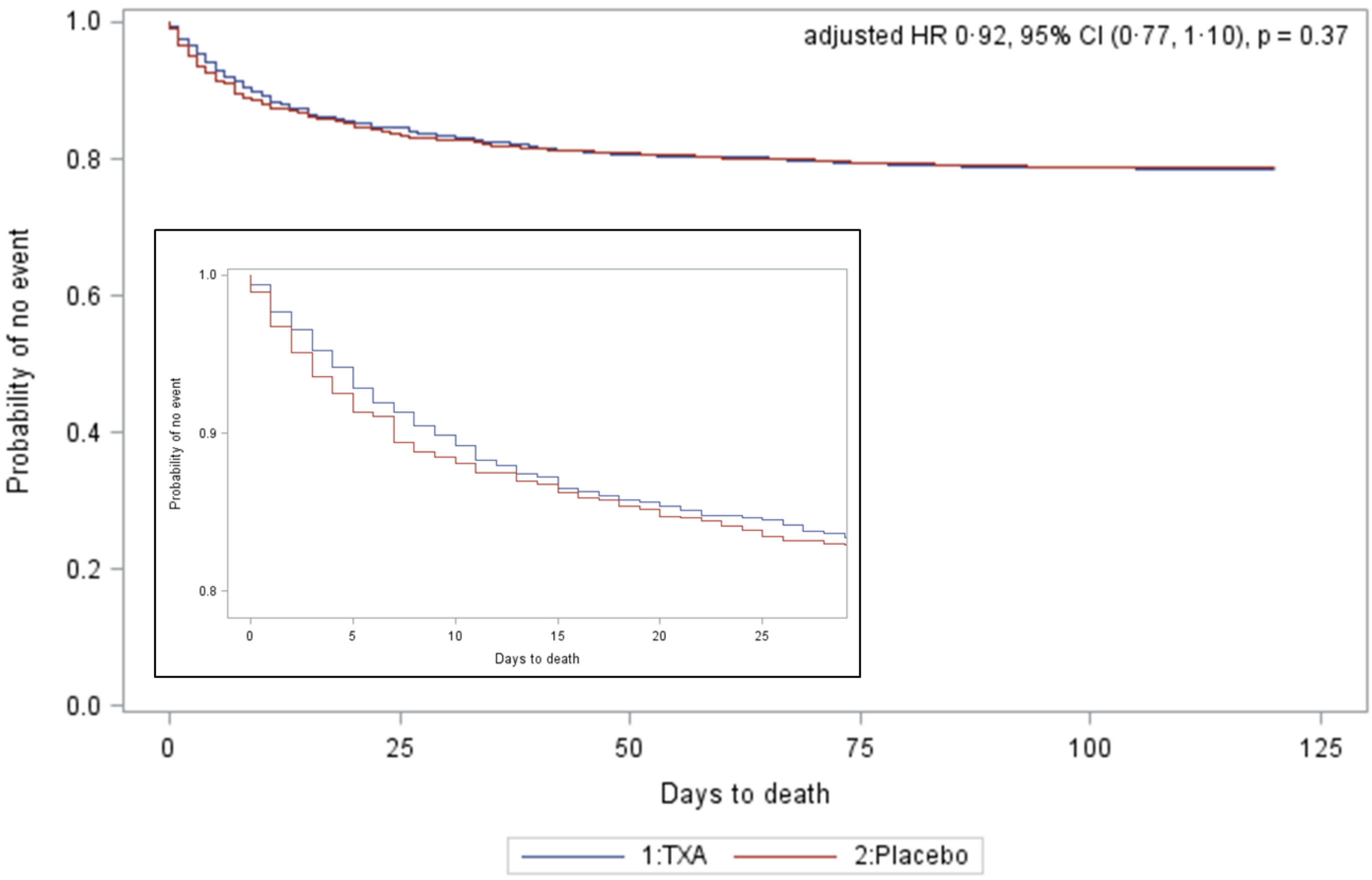

1	1161	994	950	932	919	913	911
2	1164	991	949	934	923	917	916

Writing Committee

Nikola Sprigg, Katie Flaherty, Jason P Appleton, Rustam Al-Shahi Salman, Daniel Bereczki, Maia Beridze, Hanne Christensen, Alfonso Ciccone, Ronan Collins, Anna Czlonkowska, Robert A Dineen, Lelia Duley, Juan Jose Egea-Guerrero, Timothy J England, Kailash Krishnan, Ann Charlotte Laska, Zhe Kang Law, Serefnur Ozturk, Stuart J Pocock, Ian Roberts, Thompson G Robinson, Christine Roffe, David Seiffge, Polly Scutt, Jegan Thanabalan, David Werring, David Whynes, Philip M Bath, for the TICH-2 Investigators

Protocol development

Alexandra Erven (Nottingham Clinical Trials Unit), Gillian Bumphrey (Nottingham Clinical Trials Unit), Nikola Sprigg (Nottingham), Diane Whitham (Nottingham Clinical Trials Unit)

Trial Steering Committee

Independent members: Colin Baigent (Chair, Oxford), Christine Knott (Nottingham), Yvo Roos (Amsterdam), Matthew Walters (Glasgow)

Grant holders: Rustam Al-Shahi Salman (Edinburgh), Philip Bath (Nottingham), Robert Dineen (Nottingham), Lelia Duley (Nottingham), Timothy England (Derby), Stuart Pocock (London), Ian Roberts (London), Thompson Robinson (Leicester), Christine Roffe (Stoke-on-Trent), Nikola Sprigg (Nottingham), David Werring (London), David Whynes (Nottingham)

Patient-public representative (Nottingham): Malcolm Jarvis (Oxford)

Sponsor's representative: Angela Shone (University of Nottingham)

International Advisory Committee

Denmark – H Christensen, Georgia – M Beridze, Hungary – D Bereczki, Italy – A Ciccone, Malaysia – J Thanabalan, Poland – A Czlonkowska, Republic of Ireland – R Collins, Spain – J Egea-Guerrero, Switzerland – P Lyrer, Turkey – S Ozturk

Data Monitoring Committee

John Bamford (Chair, Leeds), Martin Bland (York), Graham Venables (Sheffield), Wei Tan (Nottingham Clinical Trials Unit)

Events (outcome, SAE) Adjudicators

Timothy England (Derby), Amit Mistri (Nottingham)

Neuroimaging Adjudicators

Alessandro Adami (Italy), Lesley Cala (Australia), Ana Casado (Edinburgh), Robert Dineen (Nottingham), Rebecca Gallagher (Leicester)

Trial management committee

Senior Trial Managers: Sally Utton (2012-14), Diane Havard (2015-18)

Trial Manager: Hayley Foster (2013-16), Pauline Hyman-Taylor (2017)

UK Coordinators: Margaret Adrian (2012-17), Tanya Payne (2012-15), Alice Durham (2013-14), Harriet Howard (2014-15), Michael Stringer (2014-17), James Kirby (2015-16), Jamie Longmate (2015-17), Chris Lysons (2017-18), Nadia Frowd (2017-18)
International Coordinators: Sarah Grant (2013-14), Joanne Keeling (2014-16), James Kirby (2015-16), Robert Gray (2016-18)
Outcome Coordinators: Lyndsey Cobane (2012-14), Kathy Whittamore (2013-14), Sarah Grant (2013-14), Joanne Keeling (2013-16), Jennifer Smithson (2014-16), James Kirby (2015-16), Gemma Walker (2015-16), Hayley Gregory (2016-17), Robert Gray (2016-18), Chris Lysons (2017-18), Nadia Frowd (2017-18)
Statisticians: Lydia Fox (2012-13), Aimée Houlton (2012-13), Katie Flaherty (2013-18), Polly Scutt (2013-18)
Programming/database management: Liz Walker (2012-18), Lee J Haywood (2012-18), Richard Dooley (2013-18)
Physicians: Kailash Krishnan (2012-15), Jason Appleton (2015-18), Zhe Kang Law (2016-18)
Neuroimaging: Robert Dineen (2013-18), Stefan Pszczolkowski Parraguez (2015-18), Azlinawati Ali (2015-18), Kailash Krishnan (2012-15), Zhe Kang Law (2016-18), Adam Bischoff (2017-2018)
Trial pharmacist: Gillian Bumphrey (2012), Sheila Hodgson (2013-2018)
Data managers: Mark Sampson (2013-17), Victoria Maddox (2017-18)
Finance: Wim Clarke (2012-17), Tania Perehinec (2017-18)
Secretaries: Yvonne Smallwood (2012-18), Lauren Dunn (2013-14), Monika Kowalczyk (2014-18)
Administrators (temporary): Georgina Phillips (2013), Harry Banks (2013-14), Esther Akanya (2014), Sean McLoughlin (2015), Richard Barks (2015-17), Tierney Tindall (2016-17), Tim Logan (2017)

Participating countries, site and investigators

Denmark

Bispebjerg Hospital: A Hansen, C Ovesen, C Madelung, J Marstrand, K Aegidius, L Hajdarevic, M Folke, P Meden, S Rosenbaum

National co-ordinator: H Christensen

Telephone follow-up co-ordinators: C Ovesen, L Christensen

Georgia

Tblisi State Medical Institute: N Kakabadze, D Kakabadze, N Khizanishvili, S Mataradze, T Kherkheulidze

Tblisi Central Hospital: A Tevdoradze, N Khizanishvili, T Tsanava, T Kherkheulidze

The First University Clinic of TSMU: M Beridze, M Mdivani, N Khizanishvili

National co-ordinators: M Beridze

Telephone follow-up co-ordinators: B Tsiqarishvili, N Beridze

Hungary

Semmelweis University: D Bereczki, D Sisak, I Sipos, I Vastagh

National co-ordinator: D Bereczki

Telephone follow-up co-ordinators: D Sisak, I Sipos

Italy

ASST di Mantova: A Ciccone, A Lanari, A Magherini, F Girolami, F Notturno, G Silvestrelli, L Bertolani, M Marchini, M Tottola, P Buzzi, S Silipo

Arcispedale Santa Maria Nuova, Azienda Unità Sanitaria Locale-IRCCS Reggio Emilia: M Zedde, J Bottini

Como Sant'Anna: L Tancredi, A Sampietro, F Di Palma, L Frangi, M Arnaboldi, N Mascoli, S Vidale

Nuovo Ospedale Civile S. Agostino-Estense, Modena University Hospital: A Zini, G Bigliardi, L Vandelli, L Picchetto, M L Dell'Acua, F Rosafio

Ospedale di Branca: S Ricci, C Padiglioni, T Mazzoli

Ospedale di Città di Castello: S Ricci, A Mattioni, C Padiglioni, I Sicilia, R Condurso, S Mastrocola, S Cenciarelli

Ospedale di Rovigo: M Russo, D Fulitano, M Lucchetta, M Chinaglia, M Gentile, M Zamagni, R L'Erario

Ospedale San Giovanni Battista, Foligno: F Corea, A Guidubaldi, S Micheli

National co-ordinators: A Ciccone

Telephone follow-up co-ordinators: A Sgoifo, T Cantisani

Malaysia

Universiti Kebangsaan Malaysia Medical Centre: Z K Law, A B Azizi, A S Jaafar, B H Soon, C J Toh, F W Tajurudin, F Farizal, H J Tan, J Thanabalan, K Palaniandy, H P Tan, M I Ariff, M Omar, N Mohamed Ibrahim, R Remli, R Kumar, S Mukari, S Mat Adam, S Paramasvaran, S Sahibulddin, W Asyraf, W N N Wan Yahya

Hospital Seberang Jaya: I Looi, A Rusli, F Azizan, F Adawiyah, F Ooi, C E Loo, L K Loo, D S Marshall, P D Suppiah, S K Maniyam, S P Long

Hospital Sultanah Nur Zahirah: A Zariah, A Khairul, H Sabur, M Muda, N Mulalek

Hospital Tengku Ampuan Rahimah: U A A Ungku Mohd Zam, L Hui, N Ali, N S Isa, S H Toh, W Bee, Y Kheng

Hospital Umum Sarawak: W C Law, I Yew, M Mosen, N Sharudin, T Yian, W M Zaw (UNIMAS)

National co-ordinators: Z K Law, J Thanabalan

Telephone follow-up co-ordinator: B H Soon

Poland

Institute of Psychiatry and Neurology: A Członkowska, A Kobayashi, D Kozera-Strzelińska, E Bronisz, M Karliński

National co-ordinator: A Członkowska

Telephone follow-up co-ordinator: M Karliński

Republic of Ireland

Tallaght Hospital: R Collins, A Fallon, D Ryan, D Fitzpatrick, L W Kyaw Tun, N Cogan, R Coary, S Greene, T Coughlan

Mater Misericordiae University Hospital: S Murphy, M Marnane, P Kelly, S Coveney

Naas General Hospital: P O'Brien, A O'Driscoll, A O'Flaherty, C Byrne, M Martin, P Daly, S Mello

National co-ordinator: R Collins

Telephone follow-up co-ordinator: C Burke

Spain

Hospital Universitario Virgen Del Rocio: J J Egea-Guerrero, A J Roldan-Reina, A Rodriguez-Rodriguez, A M Ferrete Araujo, A Martinez Roldan, B Solano Hernandez, C M Rosso Fernadez, D X Cuenca Apolo, G Rivera Rubiales, L Martin-Villen, M Yaguez, M D Rincon Ferrari, M P Duran Martinez, N Palomo Lopez, R Zaida, M Yaguez

National co-ordinator: J J Egea-Guerrero

Sweden

Danderyd Hospital: A C Laska, A Grunfeldt, E Rooth, E Isaksson, H Asplund, N Greilert

Skaraborgs Hospital: B Cederin, E Bertholds, E Åkerhage, M Fantenberg

National co-ordinator: A C Laska

Telephone follow-up co-ordinator: A Franzen-Dahlin

Switzerland

University Hospital Basel: D J Seiffge, S Thilemann, A A Polymeris, M Maurer, C Traenka, U Fisch, L Hert, C Becherer, A Roesler, J Henle, M Velkopolszky, C Traenka, V Grassedonio, M Rhyner, G M De Marchis, L H Bonati, S Engelter, N Peters, P Lyrer

University Hospital Bern, Inselspital: U Fischer, S Bellwald, L Panos, P Chaloulos-Iakovidis, R von Martial, R Kurmann, A Surtmann Huguenin, M Arnold, M Kormann

Kantonsspital St. Gallen: G Kagi, A Langer, A Weber, A Muller, A Luebbert, B Felbecker, C Hock, D Uffer, J Vehoff, J Walch, L Ehrmann, M Neurology, M Dicker, M Scherrer, M Kapauer, P Balcerak, V Filipin

University Hospital Zurich: S Wegener, J van Duinen, M Haeberlin, R von Bieberstein

National co-ordinators: A Roesler, M Velkopolszky

Telephone follow-up co-ordinator: P Lyrer

Turkey

Selcuk University: S Ozturk, F Eren, G Ongun, H Ekmekci, R Aygul

National co-ordinator: S Ozturk

Telephone follow-up co-ordinator: N Yardimci

United Kingdom

Nottingham University Hospital NHS Trust: G Subramanian, A Buck, A Hedstrom, A Tittle, A K Shetty, B Jackson, C Richardson, C Gaynor, D Bester, F Shelton, G Wilkes, J Roffe, J Tomlinson, J Clarke, K Whittamore, L Daunt, L Ryan, M Godfrey, N Gilzeane, O Matias, R Keshvara, S Sheikh, S Raghunathan, S Munshi, W Sunman, Z Rose

Aberdeen Royal Infirmary: M Macleod, A Joyson, B Jagpal, B MacLennan, H Gow, J Furnace, J Irvine, J Reid, J Webster, K E Klaasen, N Crouch, P Elofuke, R Clarke, S Nelson, S Ross, S Banik, S Wilkinson, V Taylor

Addenbrooke's Hospital: E A Warburton, A Maminta-Espanol, A Day, C Farron, D Day, E B Amis (Jumilla), E OBrien, H Hayhoe, H Robinson, J Mitchell, J McGee, J Sesay, K Khadjooi, M Spillane, N Hannon, N Evans, N Butler, S Finlay, S Gargalas

Arrowe Park Hospital: R Davies, A Hill, A Dodd, B Menezes, D Lowe, D Jose, G Sangster, H Moroney, J Barrett, L Duerden, N Robinson, P Owings, P Weir, S Whittingham-Jones, S Cherian, S Downham, V Gott, V Little

Barnsley Hospital: J Humphrey, A M Ahmed, C Denniss, J Griffiths, K Kay, M K Albazzaz, S A Pearson, S Johnson

Belfast Health & Social Care Trust: E Kerr, A Fulton, A Hunter, E (Scullion) Crawford, M Kinnaird, P Fearon, S Cuddy, S Tauro

Blackpool Victoria Hospital: S Tucker, C Jeffs, E Stoddard, M Caswell, N Waddington

Bradford Royal Infirmary: H Ramadan, B Hairsine, C Patterson, I Melvin, J Greig, M Hooley, O Quinn, R Bellfield, S Maguire, W Gaba

Bucks Healthcare NHS Trust: S Al-Nahhas, A Benford, A Misra, M Burn, S MacTavish

Calderdale Royal Hospital: A Nair, J Greig, M Robinson, M I Alam, P Rana, S Prasad

Charing Cross Hospital (Imperial): O Halse, A Subanandan, A Aravind, A Sivagnanaratnam, A Kar, B Aweid, B Hazel, G Todorov, H Jenkins, K Harvey, L Gardener, M Hashmi, M Gomez-Choco, M Venter, M Rasool, M Haji-Coll, O Geraghty, P Sharma, P Wilding, R Redwood, S Mashate, S Jamil, S Banerjee, U Bojaryn, V Tilley, V Nguyen, Z Brown

Chesterfield Royal Hospital: M Sajid, M Ball, R Gascoyne, T Hendra

Colchester Hospital University NHS FT: J Ngeh, A O'Kelly (Wright), D Campbell, R Saksena, R Sivakumar

Countess of Chester Hospital: S Haider, A Nallasivan, C Perkins, H Jeffrey, K Dodd, K Chatterjee, M McLeod, S Seagrave, S Leason, S Cotgreave, T Chakraborty, T Webster

Doncaster and Bassetlaw Hospitals: D Chadha, D Walstow, G Herdman, K Smith, L Holford, M Kini, P Anderton, R Fong

Dorset County Hospital: H Proschel, A Oglesby, D Jenkinson, D Kelly, D Maitland, H Bray, L Billett, P Cook, P Williams, S Horton, S Williams (Moreton), S Sharpe, S Wilsher, S Jones

East & North Hertfordshire NHS Trust: A Pusalkar, A Cook, A Asokanathan, H Beadle, K Chan, M Massyn, P Aruldoss, P Dangri

East Sussex Healthcare NHS Trust: C Athulathmudali, A Khan, E Barbon

Fairfield General Hospital: R Namushi, D Morse, L Johnson, N Saravanan, P Jacob

Forth Valley Royal Hospital: A Byrne, A Smart, C McGhee, C Copeland, M Macleod, S Grant

Gloucestershire Royal Hospital: D Dutta, A Patel, C Hughes, D Ward, F Davis, G Whittingham, J Dalton, J Turfrey, J Russell, K Bowstead, K Collins, L Hill (Ryan), L Iddon, P Brown, S Beames, S O'Connell, T de Weymarn, T Mitchell, V Hughes

Great Western Hospital: S Haig, A Colston, C Gurney, G Gramizadeh, G Ramadurai, J McDonald, J Derham, L Matter, L Williams, M Bajoriene, S Al-Nahhas, S Kumar, S Windebank, T Slade, T Godfrey

Hull Royal Infirmary: R Rayessa, A Abdul-Hamid, E Clarkson, J Mann, P Williams

James Cook University Hospital: D Broughton, A Bergin, A Sigsworth, A Young, A Annamalai, D Tryambake, K Chapman, L Dixon, S Al Hussayni, S Whitehouse

Kent and Canterbury Hospital: H Baht, A Thomson, I Burger, P Akhurst

King's College Hospital: D Manawadu, A Karimi, A Brigden, A Kulendran, B Chitando, C Hastings, C Lovekin, E Cattermole, E McKenzie, E Owoyele, J Teo, J Good, J Lim, J Aeron-Thomas, K Marks, K Anderson, L Kalra, L Perkins, L Leonie, M Docksey, M O'Sullivan, N Sylvester, N Mazibuko, N Sikondari, O Adegbaju, P Rao, S Gargalas, S Hassan

King's Mill Hospital: M Cooper, A Rajapakse, I Wynter, K Whysall, M Nasar, S Smith

Leeds General Infirmary: S Limaye, A Hassan, A Bailey, D Waugh, E Veraque, J Britton, K Thorpe, L Makawa, L Mandizvidza, L Idrovo, M Randall, M Kambafwile, P Wanklyn, S Fry, V Papavasileiou

Leicester Royal Infirmary: D Eveson, A Mistri, C Patel, C Machili, C Stephens, K Musarrat, L Manning, M Y Lam, R Marsh, S Khan, S Anand, T Robinson, V Haunton

Luton and Dunstable: L Sekaran, D Phiri, F Justin, L Tate, S Sethuraman

Macclesfield District General Hospital: M Sein, D Leonard, E Gibson, M Naeem, R Tabbara, Y Gruenbeck

Medway Maritime Hospital: M Mamun, A Mahmood, A Woods, B Sandhu, J Nichols, J Jones, O Abimbola, R Aderinola

Morriston Hospital: Q T H Anjum, C Davies, C Clements, L Quinn, M Krishnan, M Wani, S Storton, S Treadwell

New Cross Hospital: K Fotherby, A Stevens, A Willberry, D Morgan, D Butler, K Preece, M Maw, N Ahmad, S Das, S McBride

Norfolk & Norwich: N Shinh, A Dasgupta, A Wiltshire, A Chakrabarti, C Hmu, G Ravenhill, G Rabai, J Jagger, J Keshet-Price, K Metcalf, P Sutton, S McDonald, T Salman

North Bristol NHS Trust: J Kendall, A Stockford, B Faulkner, L Rannigan, P Clatworthy, R Bosnell, R Worner, S Hierons

North Tees and Hartlepool Hospitals NHS Trust: I Anwar, C Ambulo, D Bruce, M Platton, S Crawford, T Nozedar

Northampton General Hospital: D O'Kane, F Faola, J O'Callaghan, K Smith, K Hall, L Campey, L Brawn, M Blake, P Boovalingam, S Brown

Northwick Park Hospital: D L Cohen, A Chandrakumar, A David, A Chamberlain, A Oshodi, E Owoyele, F Guo, J Devine, L Burgess, M Ngwako, M Mpelembue, P Poku, R Licenik, R Bathula, S Doubravska, V Sukdeo

Peterborough & Stamford Hosps NHS Foundation Trust: P Owusu-Agyei, I Obi, N Temple, N Butterworth-Cowin, S Subramonian

Pilgrim Hospital: D Mangion, A Hardwick, C Constantin, K Netherton, S Markova

Pinderfields General Hospital: M Carpenter, A Drake, A Stanners, A Needle, C Tatterton, D Bowes, G Bateman, H Brooke, J Archer, J Ball, L Jackson, P Datta, S Tempest

Poole Hospitals NHS Foundation Trust: S Ragab, B Wadams, C Dickson, E Jinks, J Berry, J Leggett, J Dube, L Gleave, T Garcia

Portsmouth Hospitals NHS Trust: U Sukys, A Suttling, C Thompson, C James, D Jarrett, D McFall, J Tandy, L Peck, P Howard, R Butler, S Whiteman, S Siddegowda, S Valentine, T Dobson, Y Davies

Princess Royal University Hospital: L Sztriha, B Piechowski-Jozwiak, B Chitando, B Bolca, D Ramsey, F K Chan, J Teo, J Lim, K Rhodes, M Alao, P Webster, S Tshuma, T Ajao

Queen Elizabeth Hospital: B Esisi, B Mokoena, B McCelland, M Bokhari, M Naeem, T Cassidy

Queen Elizabeth The Queen Mother Hospital: G Gunathilagan, G Thomas, G Boylin, S Jones, S Tilbey

Queen Elizabeth University Hospital: K Muir, A Welch, B Cheripelli, C Crosbie, D Kalladka, E Mclennan, F Moreton, F Savage, I Anderson, J Elliot, L Dymock, N Day, R Woodward, R Parakramawansha, S Baird, S El Tawil, S Ramachandran, T Hopkins, W Smith, X Huang

Queen's Hospital: S Andole, C Plewa, K Dunne, M Krommyda, N Gadapa, R Patel, S King, S Choudhary, W Wunna

Queen's Medical Centre: F Coffey, A Bolland, B Pope, C Gough, C Woodford, I Peel, I Quirk, J Risley, J Thomas, J Shepherd, J Bradder, J Warren, L Conner, L Ryan, P Dykes, P Miller, S Hodge, S Finucane

Rotherham NHS Foundation Trust: S Punnoose, J Okwera, J Harris, J Howe, S Besley, V Borkar

Royal Berkshire NHS Foundation Trust: A van Wyk, E Bowley, E Flossmann, F Jeddi, G Grimwood, G Lumsdale, J Buick, J Foxton, K Keating-Fedders, K Nagaratnam, L Keating, N (Bhupathiraju) Mannava, N Mistersky, N Haque, R Carson, R de Caux, R Slater, R Tan, T Samakomva

Royal Bournemouth & Christchurch Hospitals: B Jupp, A Iqbal, B Longland, C Bagnall, C Ovington, D Jenkinson, D Tiwari, E Rogers, G Hann, J Bell, J Roberts, M Ricciardi, M Keltos, O David, S Loganathan

Royal Derby Hospital: T England, A Hedstrom, J Scott, J Beavan, J Clarke, M Maddula, R Donnelly, S Chohan, S Zaya, E Melikyan, M Parkin, M Blair, S Keane, S Johnson, H MacKenzie, A Costa, B Rushton, C Hyde

Royal Devon and Exeter NHS Foundation Trust: J Sword, A Bowring, A Goff, A Hemsley, C Ostrowski, C Roughan, G Jennings, H Kingwell, J Kubie, J France, J Butchart, J Cageao, J Short, K Gupwell, K Miller, K Thorpe, M James, N Chivers, P Mudd, R Partridge, S Elyas, S Keenan, W D Strain

Royal Hampshire County Hospital: N Smyth, C Eglinton, D Dellafera, E Giallombardo, J Wilson, J Duffy, L Sykes, M Hancevic, Z Arfeen

Edinburgh Royal Infirmary: R Al-Shahi Salman, A Barugh, B Gallagher, B Colam, F Doubal, F Morrow, J Perry, J Coyle, J Grahamslaw, K McCormick, K Simpson, M Macleod, M S Dennis, M Reed, M Masson, N Hunter, N Arulraj, P Taylor, P Black, R O'Brien, R O'Brien, S Burgess, S Hart, T Zahoor, T Elder-Gracie, W Rutherford, W Whiteley, F Perks, C Byrdon

Royal Lancaster Infirmary: P Kumar, A Ijaz, C Thomas, C Culmsee, J Craig, L Dunn, S McBride

Royal Liverpool Hospital: P Fitzsimmons, G Fletcher, P Lopez, P Cox

Royal London Hospital: T Harrison, A A Salek-HAddadi, A Sivagnanartnam, C Dowty, D Hove, E Quelch, E Erumere, G Grimwood, G Bellhouse, G Auld, H Syed, I Pope, I Skene, J Pott, J Pincott, J Richards, K Saastamoinen, K Gunn, L Cuenoud, L Howaniec, L Argandona, L Dennis, M Chiriac, N Stewart, O Boulton, O Redjep, P Gompertz, P Daboo, R Duncombe-Anderson, R Yadava, R Erande, R Michael, R Icart Palau, S Al-Saadi, S Amlani, T Harris

Royal Preston Hospital: H Emsley, A McLoughlin, A Cullen, B Gregary, S Ahmed, S Philip, S Punekar, S Raj, S Sultan, Z Subhani, D Doyle

Royal Stoke University Hospital: R Dharmarajah, A Barry, A Butler, A Remegoso, A Morris, A Warusevitane, C Lambert, C Causley, C Roffe, C Bosanko, F G Alipio, G K Muddegowda, H Maguire, I Massey, I Natarajan, J Chembala, J Grocott, J Hiden, J Norton, K Hunter, K Finney, K Castro-Foskett, N Ahmad, N Abano, P Ferdinand, R Carpio, R Sanyal, R Varquez, R Hall, R Kinston, S Stevens, S Lyjko, S Huda, T James, U Ghani

Royal Sussex County Hospital: I Kane, A Pitt Ford, D Hansen, D Mullan, E Barbon, F Caddye, J Breeds, L Behar, L Latter, L Ortiz-Ruiz de Gordoa, N Gainsborough, P Thompson, S Hervey

Royal United Hospital Bath NHS Trust: S Al-Nahhas, A Cross, A Wedgwood, B Madigan, C Hardy, C Peter, C Taylor, D Watson, D Howcroft, D Button, D Williamson, E Day, E Brown, E Gilby, E Carver, F Beech, H Newton, J Choulerton, J Jones, J Avis, J Bareham, K Peacock, K Manning, L Shaw, L Gbadamoshi, M Price, N V Kumar, N Adams, N Jakeman, P Paterson, P Kaye, R Porada, R Oaten, R Furse, S Nushaj, S McCann, S Lucas, T Slade

Royal Victoria Infirmary (RVI): A Dixit, A Barkat, A Gani, A Dyker, C Hubbuck, C Hays, C Tunnell, C Stevenson, E Horsley, E Elkin, G Ford, G M Smith, H Curry, H Guy, J L Byers, J Bird, J Davis, K Storey, L Giraldo, L Finlay, M Davis, M Fawcett, M Hossain, S Dima, S Louw, S Woodward, S Crawford, T Thompson, V Hogg

Russells Hall Hospital: S Kausar, A Salam, A Cole, A C Gregory, A K Banerjee, C Allcock, D Beckley, L Kadiki, M Maw, M Rasappan, R Gidda, S Fullwood, S Merotra

Salford Royal Hospital: C Smith, A Parry-Jones, A Ahmad, A K Kishore, A Hall, A Ingham, A Suman, D Sen, E Campbell, J Barker, J Perez, J M Morell, J Kallingal, K Shaw, L Harrison, M Massyn, M Punter, M Johnes, R Jarapa, S Marshall, S Arshad, T Marsden, V Ellison, V O'Loughlin, Y Pai, Z Naing

Salisbury District Hospital: T Black, A Anthony, C Clarke, D Mead, R Fennelly

Scarborough General Hospital: J Paterson, E Temlett, K Deighton, L Brown, S Dyer

Sheffield Teaching Hospitals NHS Foundation Trust: K Harkness, A Ali, SC Blank, C Kamara, C Doyle, D Sokhi, E Richards, G Dunn, J Howe, J Janbieh, K Gill, K Dakin, M Meegada, M Randall, M Khan, R Lindert, S Bell, S Duty

South Devon Healthcare: B Bhaskaran, A Saulat, G Ayres, H Shiels, H Bearne, J Buxton, J France, K Horan, P Fitzell, S Szabo

South West Acute Hospital: B Keegan, C Blair, J Kelly, M Doherty, S Grimes

Southampton General Hospital: N Weir, R Marigold, A Blades, A Walters, B Watkins, C Cox, C Allen, D Whittaker, E Battersby Wood, F Smith, G Howard, I Gartrell, J Mitchell, L Sykes, M Brown, P Crawford, R Creeden, S Egerton, S Smith, S Evans, V Pressly

Southend University Hospital NHS Fdt Trust: P Guyler, A Muhammad, A Siddiqui, A Siddiqui, C Hmu, D Ngo, D Sinha, K X Ng, M Ramjee, N Menon, R Prabakaran, S Alam, S Tysoe, S Kelavkar, S Rashmi, S Kunhunny, S Shah, T Loganathan

Southern Health & Social Care Trust: M McCormick, C Douglas, D Craig, M Goggin

St George's Healthcare NHS Trust: B Clarke, A Blight, A Thompson, A Dawson, A Pereira, A Nitkunan, B Moynihan, B Patel, C Lovelock, C Medcraft, C Watchurst, C Loughran, C Lambe, C Orefo, F Watson, G Cluckie, K Kennedy, K Kong, K Moodley, L O'Duffy, L Zhang, L Young, M Yogarajah, N Jeyaraj, N Clarke, N Gilliat, N Chopra, N Dayal, P O'Mahony, R Williams, R Ghatala, S Shribman, S Rounding, S Trippier, T Adedoyin, T Hardiman, U Khan, V Jones

Stepping Hill Hospital: J Vassallo, A Khan, A Hasan, D Morse, D Leonard, H Cochrane, J Curtis, J Awolesi, K Dizayee, M Malik, N Mumtaz, S Krishnamoorthy, S Qamar

Sunderland Royal Hospital: J O'Connell, B Mokoena, A Smith, C Fox, D Gulliver, E Osborne, M Bokhari, M Myint, R O'Brien, S Crawford

Taunton & Somerset NHS Trust: R Whiting, C Pawley, E Foster, E J Homan, H Durman, J Foot, L Foote, L Brotherton, M Hussain, M Harvey, P Baines, S Brown

The County Hospital: C Jenkins, F Price, J Powell

The Ipswich Hospital NHS Trust: M Chowdhury, A Newman, A Mackett, A Salih, D Hartin, D Beeby, G Rose, H Blaylock, J Dorward, J Calleja, L Hazlehurst-Brooks, L Hunt, M Ibraheem, R Ulph, S Alam, S Race, S Das, S Brixey, W Davison

Queen Elizabeth Hospital Birmingham: D Sims, C Sutton, E Littleton, E Hardy, J Cunningham, J Hurley, J McCormack, J Khaira, M Wilmot, M Bates

The Royal Surrey County Hospital: K Pasco, A Blight, B Olga, C Peixoto, G Longwe, J Corns, S Bradfield

United Lincolnshire Hospitals Trust: M Soliman, C Hewitt, I Wahishi, J Sharma, J Hindle, R Brown, S Arif, S Leach, S Butler

University College London Hospitals NHS Fdtn Trust: D Werring, A Rafique, A Ashton, A Sivagnanartnam, A Shukla, A Banaras, B Athwal, C Hogan, C Watchurst, D Burrage, D Epstein, D Turner, D Austin, E Elliott, I Jones, J Pleming, K Patel, L Benjamin, L Howaniec, L Latter, L Potter, L Ginsberg, L Crook, M Bhargava, M Brezitski (Abramova), M Sayed, N Francia, N Oji, R Erande, R Perry, R Luder, R Laird, S Al-Saadi, S Mufti, S Feerick, S Tshuma, T Yates, T Enes

University Hospital Aintree: T Hlaing, C Cullen, D Shackcloth, J Peters, R Kumar, R Durairaj, T Fluskey, V Sutton, Z Mellor

University Hospital of North Durham: D Bruce, B Esisi, E Brown, G Shim, G M Smith, G Rogers, K Z Nyo, M Dhakal, S Dima, S Clayton, Y Pai

University Hospitals Coventry & Warwickshire NHS Trust: S Joshi, A Bwalya, A Kenton, B Dallol, B Hunt, H Hassan, I Martin, M Sakr, S Nyabadza

Victoria Hospital: V Cvoro, A Kinnon, K McCormick, K Ullah, M Couser, M Carew, N Hunter, N Chapman, S McAuley, S Pound

Watford General Hospital: D Collas, E Walker, M Bhandari, S Sundayi, S Gill

West Suffolk Hospital: A Nicolson, A Azim, D Singh, E Stebbings, J Imam, J White, L Wood, M Krasinska, S Alam

William Harvey Hospital: T Webb, A Verrion, A Thomson, D Hargroves, E Beranova, H Rudenko, I Balogun, L Cowie, S Walker, T Cosier

Worthing Hospital: N Sengupta, C Dewar, J Margalef, M Metiu

Yeovil District Hospital: K Rashed, C Smith, C Buckley, D Wood, L Balian, N Marks, S Board, T Pitt-Kerby

York Hospital: P Wanklyn, C Anazodo, J Coyle, K Deighton, L Doughty, M O'Neill, M Porteous, M Keeling, N Dyer, P Willcoxson, P Clark-Brown, P Inns, R Mir
